# Cross-cultural, age and gender validation of a computerised questionnaire measuring personal, social and environmental associations with children's physical activity: the European Youth Heart Study

**DOI:** 10.1186/1479-5868-5-29

**Published:** 2008-05-19

**Authors:** Yngvar Ommundsen, Angie Page, Po-Wen Ku, Ashley R Cooper

**Affiliations:** 1 The Norwegian School of Sport Sciences, Oslo, Norway; 2 University of Bristol, Department of Exercise, Nutrition and Health Sciences, Bristol, UK; 3 National Changhua University of Education, Graduate Institute of Sports and Health, Changhua City, Taiwan

## Abstract

**Objective:**

This study investigated the construct validity of a computerised self-assessment tool to measure psychological, social and environmental influences of young peoples' physical activity. First, analyses of the measure's factorial validity, invariance across, age, gender culture were conducted. Second, the ability of the derived subscales to discriminate between children representing different levels of self-reported and objectively measured physical activity behaviour was examined.

**Methods:**

Participants were 1875 boys and 2078 girls (total = 3958) aged 9–10 years (n = 1955, mean age = 9.65 ± 0.42) and 15–16 years (n = 2003, mean age = 15.49 ± 0.50) from four European countries in Northern, Southern and Eastern Europe who took part in the European Youth Heart Study (EYHS). Children completed the computerised self-assessment tool with support from the researcher if requested. Self-reported exercise and an objective measure of physical activity (Actigraph model 7164) were used for additional construct validation purposes.

**Results:**

Overall evidence of good fit indicating satisfactory factorial validity and cross-cultural, age and gender invariance for 3 of the 4 measurement models were obtained. The majority of measures were also significantly different for those with high versus low levels of physical activity.

**Conclusion:**

Overall, the computerised questionnaire holds promise for use cross-culturally with male and female children and adolescents to measure perceived personal, social and environmental influences on physical activity. Further development of the measures pertaining to perceived environmental influences seems warranted.

## Background

Optimal physical activity levels are suggested to be an important factor in the development and/or maintenance of childhood health [1-3]. Health benefits associated with physical activity include healthy growth and development, improved psychological well being and maintenance of energy balance [2,4,5]. Knowing the influences on physical activity in young people is an important first step in designing physical activity intervention programmes to increase physical activity levels in children and adolescents [6]. This is important as experiences of physical activity in childhood may impact on subsequent participation as an adult [2]. The strength of 'tracking' of physical activity is equivocal but is generally reported to be moderate throughout childhood and weaker from childhood to adulthood [7].

Several factors have been described when trying to understand influences on physical activity among children and youth. Three sets of influences embedded in social cognition approaches and ecological models of behaviour have gained repeated support in the physical activity literature. These three sets comprise 1) ***personal factors ***reflecting *outcome expectations *emanating from social-cognitive theory and attitude-behaviour models, and *competence perceptions and enjoyment *emanating from White's model of effectance motivation embedded in Harter's competence motivation theory; 2) ***social factors ***reflecting *social support from significant others *emanating from Bandura's social-cognitive theory; 3) ***physical and social environmental factors ***reflecting *physical opportunities for physical activity, access to safe environments and social restrictions *emanating from Bandura's social-cognitive theory and social-ecological models of physical activity behaviour [8,9]. In his conceptual Youth Physical Activity Promotion model based on the Precede-Proceed framework, Welk [9] categorized the above three sets of influences as representatives of predisposing, reinforcing, and enabling factors respectively [9]. These three sets of perceived personal, social and physical and social environmental factors seem to hold great promise for understanding physical activity behaviour [9,10]. Other factors have received less attention, e.g. few measures have been investigated related to teachers as potentially influential on young peoples' physical activity [6,8]. Nevertheless, Welk in his model [9] included social support of teachers as significant others' social support under the umbrella of reinforcing factors, so including teachers as a source of social support alongside e.g. parents, peers seems reasonable. Recent research highlighting the importance of context specific activity e.g. active commuting and informal games at school supports the focus on the school and the potential role of teachers to facilitate or inhibit activity related perceptions and behaviour [11].

Despite existing efforts to determine perceived personal, social and environmental influences of physical activity behaviour in children [8], estimates of construct validity of potential correlates are still limited [6,8,11,12]. First, more evidence of the factorial validity and gender, age and cross-cultural invariance of correlates is needed. Second, few studies have included a range of measures of perceived environmental influences within the same sample to reflect social-ecological models [8]. Third, there is a lack of studies that have examined the ability of perceived personal, social and environmental factors to discriminate between children's objectively measured physical activity levels [13-15].

In terms of factorial validity and estimates of invariance, few studies have used psychometrically robust latent variables in investigating influences on physical activity [13,15], and despite the increase in large multi-centre studies few confirm the validity of measures across different languages or cultures [15]. Examining cross-cultural, age and gender invariance is important to be confident that any differences in reported regional, cultural or population based scores are not merely a function of differences in interpretation of the measures according to context, language, age or gender. There is a possibility that the responses to items may be coloured by a particular cultural norm within a country, an age cohort or a specific gender [16,17]. The cross-cultural psychometric integrity of sport and exercise psychology measures can be examined using structural equation modelling to test factorial invariance (17). Investigating factorial invariance across countries, age and gender groups allow us to examine whether a questionnaire measures a latent construct similarly across samples. It involves a comparison of the equivalence of the variance- covariance matrix, factor structure, factor loadings, factor variance and item uniqueness across subgroups.

Perceived personal, social and environmental influences are difficult to measure, particularly in young people [13]. Interview methods are time-consuming, and secondary data collection via the parents is open to bias [18]. Traditional paper and pencil self-report measures are also problematic due to language and cognitive limitations in this age group [19]. Situational factors may also impact on the quality of data obtained from self-report questionnaires, such as variation in instructions given and how they are interpreted by respondents. Furthermore problems in use of language and content relevance can impact on responses to questions and the predictive ability of the determinants in different groups. Overcoming these barriers is critical for multi-centre studies which seek to combine and compare data from different populations. Computers offer a potential medium for helping to reduce the impact of these problems [19,20]. Use of computers for data collection may improve the reliability and validity of self-report measures by: a). Providing a stimulating and familiar format for the majority of children with comprehensive instructions incorporating sound, images and text to facilitate understanding of the questions; b) Ensuring all questions are completed and unreasonable responses are not accepted, so reducing missing data; c). Incorporating triangulation techniques to systematically cross-check children's responses; d). Entering data concurrent with administration, so reducing error; e). Providing standardised instructions minimising potential variability in administration and f). Providing an anonymous environment for children to respond to, limiting the potential bias of interaction with an adult. For these reasons a computerised questionnaire measuring perceived personal, social and environmental correlates of physical activity and children's health was developed and validated in a UK sample [21,22].

### Main aim

The main aim of the current study was to investigate the construct validity of measures of three (e.g. personal, social and physical and social environmental) sets of perceived influences of physical activity behaviour in children and adolescents from four European countries. To this end a two-step procedure was used. First we examined the measures with respect to factorial validity and their invariance according to country, age and gender. Second, we examined whether the measures tested in step one were able to discriminate between children with different levels of self-reported and objectively measured physical activity behaviour.

## Methods

### Participants

Data from participants included in the European Youth Heart Study (EYHS) were used. The EYHS is a multi-centre, international study addressing the determinants, prevalence and aetiology of cardiovascular disease risk factors in children aged 9 and 15 years across four culturally different European countries [23]. Boys and girls aged 9 and 15 years old were randomly selected to participate from study locations in Northern Europe (Norway: Oslo and Denmark: Odense), Southern Europe (Portugal: Madeira) and Eastern Europe (Estonia: Tartu). All locations carried out the same protocol. Full details of the overall study design, sampling procedures and participants are provided in Riddoch and co-workers [23]. The age groups representing 9 and 15 years old were chosen to broadly represent children on either side of puberty. At each study location, a defined population of minimum 1000 boys and girls ages 9 and 15 y were recruited. Samples of children were drawn in a similar fashion within each study location. At each location, a sampling frame of schools using official lists was compiled. The primary sampling units were schools and the secondary units were student registers or roll calls. Schools were stratified by the socio-demographic characteristics of their local areas. Each school was given a weighting according to the number of children enrolled and a minimum of 20 schools were randomly selected using probability proportional to school size. Children of appropriate ages were sampled randomly (random number tables) within schools using the school register by means of a two stage cluster sampling procedure. This sampling procedure secured a) a similar overall participation proportion in each country (73–76%), and b) that the participation proportions were reflective of the populations at each study locations with respect to gender, ages, racial/ethnic status and socio-demographic background [1]. In total, 1875 boys and 2078 girls (total = 3958) aged 9–10 years (n = 1955, mean age = 9.65 ± 0.42) and 14–15 years (n = 2003, mean age = 15.49 ± 0.50) participated.

### Measures

#### Correlates of physical activity

The computerised questionnaire contained measures designed to capture theoretically derived relevant constructs identified as perceived personal, social and social & physical environmental influences on physical activity in young people. See Table 1 for details of all measures (scales and subscales with respective items based on the confirmatory factor analyses results). Items of each subscale were summed and divided by number of items per subscale to represent the mean score for the following constructs:

**Table 1:** Summary of perceived personal, social and environmental measures based on confirmatory factor analysis

**Measures including scales and subscales with respective items and response formats from computerised questionnaire**	**Source of original scales**
**Enjoyment-Competence scale (response format 1–3)**	Brustad^(1993)^
*Subscale Enjoyment*	Harter^(1978)^
I have more fun playing games and sports than doing other things	
Playing games and sports is the thing I like to do best.	
I wish I could play more games and sports than I get chance to.	
I usually prefer to watch rather than play games	
I really like doing PE at school.	
*Subscale Competence*	
I feel that I am better than most other kids my age at games and sports.	
I feel really bad when I get out of breath from running around	
I feel that I can easily keep up with other kids when playing games and sports.	
**Outcome expectations scale (response format 1–3)**	Trost et al^(1997)^
*Subscale Social Expectations: If I were to exercise most days it would*....	
Be fun	
Help me make new friends	
Help me be with my friends more	
Help me look good to others	
*Subscale Functional Expectations: If I were to exercise most days it would*....	
Get or keep me in shape	
Make me better in sports	
Help me be healthy	
Help me control my weight	
Give me energy	
**Perceived social support scale (response format 1–4)**	Sallis et al.^(1999)^
*Subscale Parental Support: How often does your mum or dad*...	
Take you to exercise or play sports	
Watch you take part in exercise or sports	
Exercise or play sports with you	
*Subscale Parental Encouragement*: *How often does your mum or dad*...	
Tell you to exercise or play sports	
Tell you that exercise is good for your health	
*Subscale Peer support*	
How often do your friends exercise or play sports with you?	
How often do you ask your friends to play out with you?	
How often do your friends ask you to play out with them?	
*Subscale Teacher support: How often does your teacher*....	Items for teacher support generated through pilot study
Talk about exercise in lessons	
Organise or play games with you apart from PE	
Tell you to exercise or play sports	
	
**Social-physical environment scale (response format 1–3)**	Sallis et al.^(1999^
*Subscale opportunity*:	Trost et al^(1997)^
It is safe to walk or play alone in my neighbourhood during the day.	
There are other children near by home to go out and play with	
There is somewhere at home I can go out and play	
*Subscale Facility:*	
There are playgrounds or parks close to my home where I can play	
At school there are playgrounds or fields where I can run around	
*Subscale License:*	
I always have to tell my parents where I am when I go out	
If I am going out I always have to be back by a certain time	

1. *Perceived competence-enjoyment *comprising two subscales, competence and enjoyment.

2. *Perceived outcome expectations *comprising two subscales; functional outcome expectations and social outcome expectations.

3. *Perceived social support *comprising four sub-scales, parental social support, parental encouragement, peer support, and teacher support.

4. *Perceptions of the social-physical environment *comprising three sub-scales, opportunity, facility and licence.

[8,9,24,25]. With the exception of the teacher support, all measures consisted of items originating from previous research, where evidence of their reliability and validity has been demonstrated [6,26-28]. All measures were collectively pilot tested on a smaller sample of English children from Bristol and London. The younger children were selected for pilot testing as these were most likely to find the questions cognitively challenging. Six focus groups were initially carried out with groups of 6–8 children of mixed gender in Bristol. Where measures in the format of existing scales were available the items were discussed with children to determine their understanding of what the items were assessing and their ability to differentiate between the responses. For items where existing measures were unavailable (e.g. perceived teacher support) open questions were used and items generated after content analyses of responses provided by the children. In some instances items and responses were slightly modified based on feedback from the focus groups. For example, dichotomous yes/no responses were replaced with 3 to 5 point response formats. In order to keep responses age appropriate and simultaneously consistent across age groups, no responses exceeded 5-point formats. Variables with a 3-point response format ranged from 1 (definitely no) to 3 (definitely yes), variables with a 4-point response format ranged from 1 (hardly ever or never) to 4 (every day) [21]. In addition, extra items were added to reflect a broader range of perceived environmental indices than those usually studied, such as license; that is parental permission for child to play and stay out alone or with peers in the home surroundings or elsewhere [6,28]. Further validation work included a pilot test of the items on 120 Year 5 (aged 10–11 years) boys and girls from London. Test-retest reliability for the derived scales ranged from r = 0.64 to r = 0.86 (p < 0.05). All measures comprised at least three to five items to represent each construct [29]. To keep the questionnaire length to a minimum, factor analytic procedures in the pilot phase were used to determine items with the highest loadings and eliminate others based on significant cross-loading or non-loading on intended factors.

#### Physical activity behaviour

In order to examine the ability of the correlates to differentiate between children representing different levels of physical activity behaviour two measures of physical activity were used. The first was self-reported 'Stage of change for physical activity'. Studies [30] based on work by Prochaska and co-workers typically formulate five stages of readiness for change in physical activity ranging from stage one; "I am currently not physically active and I do not intend to engage in physical activity in the next 6 months" (Pre-contemplation) to stage five "I am currently regularly physically active and have been for more than 6 months" (Maintenance). The Stage of Change construct is able to differentiate between people as being able to differentiate between people with different levels of physical activity in different age groups, where those in stages 4 and 5 are more frequently physically active than those in stages 1 and 2 [31]. The second measure of physical activity was an objective measure using the MTI accelerometer (model 7164; former Computer Science Applications). The MTI is an electronic motion sensor comprising a single plane (vertical) accelerometer. The monitors are small (4,5×3,5×1,0 cm) and light (about 43 g) and are worn in an elastic belt around the waist. Verbal and written instructions were given to both the children and their parents regarding its use. All children were instructed to wear the monitor continuously during the day, except when doing water-based activities. The monitors were pre-programmed to start recording at 0500 on the first day of measurement. The activity counts detected by the accelerometer were averaged and stored every 60s for 4 consecutive days, including 2 weekdays and 2 weekend days, and the number of minutes per hour of moderate or greater intensity physical activity (> 3 METS) was calculated using established age-specific cut points [32] to divide children into high versus low physical activity groups. These groups were based on whether children met current physical activity guidelines, i.e. achieving average of at least 60 minutes of accumulated moderate intensity activity per day [3]. However as physical activity was not measured for seven consecutive days in this study, the high activity group were those who achieved at least 60 minutes per day of moderate or greater intensity for a minimum of three days. Only children with at least 10 hours of recorded time per measurement day were included in the analysis and data reduction was carried out using established macros in line with previous analyses [1].

### Procedure & ethical issues

A standardised research protocol for the EYHS was followed in collecting, processing and analysing data [23]. Study protocols conformed to the international guidelines on biomedical research and each research team complied with the ethical procedures of that country. Written, informed consent was obtained from the child's parent or legal guardian after they were given, in writing a full explanation of the aims of the study, its possible hazards, discomfort, and inconvenience. In addition, children had all the procedures verbally explained to them, together with any possible discomfort they might encounter and were given the option to withdraw at any time. Data was collected in small groups of 8–10 children throughout the school year. As part of the data collection 'day' each child completed the computerised questionnaire measuring personal, social and environmental correlates with children's health. Completion of all measures on the computerised questionnaire took on average 20–30 minutes with children left alone to complete their responses but with a researcher nearby if they needed help. The computerised questionnaire was designed to encourage considered answers and children could not proceed to the next page without completing the relevant answer. Information was obtained in advance within each school from school staff with respect to potential differences in computer competence and experience among the children. Generally, pupils' computer experience and competence were reported to be at a level that would not interfere with the quality of the data collection. Feedback from the research personnel at all four study centres confirmed this impression.

### Data analysis

#### Factorial validity and multi-group invariance for different countries, genders and age groups

To examine factorial validity, four baseline models were established for each country group respectively by confirmatory factor analyses (CFA) using EQS 6.1. Baseline models were built on the respective theoretical perspectives to which the constructs theoretically belong. [8,9,24,25]. Hence, a Competence-Enjoyment model based on the reasoning emanating from White's effectance motivation theory embedded in Harter's theory of competence motivation [9] comprised Model 1. Model 2 comprised an outcome expectation model based on Bandura's social cognitive theory [7-9], Model 3 comprised a perceived physical-social environment model based on Bandura's social-cognitive theory and social-ecological models of physical activity behaviour [7-9]. Model 4 comprised a measurement model consisting of three sub-dimensions of social support, each dimension reflecting three different sources of support (i.e. parental, peers, teachers) [9].

The exploratory factor analyses revealed patterns of factor loadings consistent with theoretical expectations. Thus, in the exploratory analysis of Model one, two factors emerged to represent the perceived competence items and perceived enjoyment items respectively. Model 2 related to outcome expectations had two factors comprising functional and social outcome expectations. In the exploratory analysis of Model 3, items pertaining to perceived parental support, parental encouragement, peer support, and teacher support, respectively, loaded on three separate factors. Finally, the items pertaining to the perceived physical-social environment model came out as three dimensions in the exploratory analysis of Model 4. These dimensions with respective items were then ordered according to personal, social and physical-social environmental sets of influences and theoretical reasoning into four separate measurement models in the confirmatory factor analyses.

Hence Model 1 represented measures of perceived personal influences that comprised an enjoyment sub-scale and a competence sub-scale; Model 2 represented measures of perceived personal influences that included an outcome expectations scale; Model 3 represented measures of perceived social influences that comprised three separate sub-scales; perceived parental support, peer support and teacher support. Finally, Model 4 represented measures of perceived physical-social environmental influences comprising a scale consisting of items pertaining to various aspects of the environment such as opportunity, license and facility.

After assessing factorial validity, factorial invariance across different samples for country, gender and age were performed respectively. The invariance of three nested models across different populations was explored. Of the three nested models, the configural model is the least restrictive, followed by 'the measurement model', then the most restrictive 'structural model'. The procedure for testing multi-group invariance using covariance structures was adopted [33]. First, the initial step for testing 'configural model' only designates that the same numbers of factors and factor-loading pattern be equivalent across four samples. The four configural models required no equality constraints imposed on the parameters. Each time, one multi-group analysis incorporates one baseline model for four groups within the same file and allows for testing invariance tests simultaneously. Invariance holds if the multi-group exhibits an adequate fit. Second, the testing for 'measurement model' addresses the issue of equality regarding to the measurement model, which requires the invariance of factor loadings and measurement error variances-co-variances by imposing on equality constraints. Byrne [33] claimed that invariance holds if the multi-group exhibits an adequate fit (besides the goodness-of-fit indices for CFA, the changes in the CFI value between nested models should be negligible, i.e. changes in CFI less than 0.01). If the recommendation cannot be met, partial measurement can be applied, which implies that all previously imposed equality constraints remain except those identified to be non-invariant. The employment of partial measurement invariance hinges on the number indicator for assessing each latent construct and at least one invariant measure remaining non-invariant measures (besides the one fixed to 1.0 for model identification) [33]. Finally, testing for 'structural model' has all the parameters to be constrained to be equal across groups, comprising factor co-variances.

A range of indices were used to examine the four models. As well as adopting the widely used maximum likelihood (ML) estimation, Satorra-Bentler Scaled χ^2 ^(S-B χ^2^) was also employed due to the concern of non-normality [34]. The recommended goodness-of-fit indices include CFI (comparative fit index) greater than 0.90; the point estimate of RMSEA (root mean square error of approximation) less than 0.05 and the 90%CI of RMSEA less than 0.08. SRMR (Standardised Root Mean Square Residual) should also be less than 0.08 [33,34]. Procedures for testing factorial invariance across different genders and age groups were conducted sequentially.

#### Reliability and further examination of validity

The reliability of sum-scores of items underlying each latent scale and subscale was examined using Cronbach's alpha scale analysis. In cases when the latent sub-scales came out as two item-measures in the confirmatory factor analysis spearman correlation was used. For additional construct validation purposes, a multivariate analysis of variance (MANOVA) was conducted to examine whether correlates significantly differed in mean values across stages of change for physical activity [30]. In line with previous research [30,31], we expected significantly higher mean scores on the measures of both personal, social, and social-environmental correlates with increasing stage of change in physical activity. Second, an independent sample t-test was performed to examine whether physical activity correlates differed in the high versus low physical activity behaviour groups. The high versus low groups were based on whether children were found to achieve versus not achieve at least 60 minutes of accumulated moderate physical activity on at least three days.

## Results

### Factorial validity and multi-group invariance

The Confirmatory factor analyses indicated that the four baseline models for all groups (country, gender and age) all yielded satisfactory good fit to the data [35] except one. In testing for the baseline Model 1 (enjoyment and competence) for four groups, the analyses of the modification indices (e.g. Lagrange Multiplier Test, LM Test) led to the specification of an error covariance between items Enjoyment "I usually prefer to watch rather than play games" and Competence "I feel really bad when I get out of breath from running around". This improved its goodness-of-fit for the perceived competence and enjoyment sub-scales [34].

As shown in table 2, the analysis of factorial invariance for all models for gender yielded satisfactory results which met the suggested criterion and without undergoing significant model modification. The outcomes of testing invariance for different age groups are shown in Table 3. The fit indices for the perceived enjoyment and perceived competence sub-scales and the four perceived social support sub-scales (including also a sub-scale labelled parental encouragement) were adequate. The structural invariance of the functional and social outcome expectation sub-scales was close to the recommended criterion (Change in CFI = 0 .02) but the perceived social-physical environment sub-scales did not meet the guideline (Change in CFI = 0.03).

**Table 2:** Tests for Factorial Invariance of the Four Models across Different Genders: Goodness-of-Fit Statistics

Model	S-B χ^2^	Df	CFI^a^	Changes in CFI^a^	RMSEA (90%CI)^a^	SRMR^a^
**Enjoyment-Competence Model**						
1: Configual model	131.55	33	0.94		0.05(0.04–0.06)	0.04
2: Measurement model	169.73	40	0.93	0.01	0.05(0.04–0.06)	0.04
Partial measurement model^b^	154.79	38	0.94	0.00	0.05(0.04–0.06)	0.04
3. Structural model	163.66	39	0.93	0.01	0.05(0.04–0.06)	0.04

**Outcome expectation Model**						
1: Configual model	168.42	49	0.94		0.04(0.04–0.05)	0.04
2: Measurement model	179.11	56	0.93	0.01	0.04(0.03–0.05)	0.04
3. Structural model	181.28	57	0.93	0.01	0.04(0.03–0.05)	0.04

**Social Support Model**						
1: Configual model	237.92	74	0.98		0.04(0.03–0.04)	0.03
2: Measurement model	248.82	81	0.97	0.01	0.04(0.03–0.04)	0.04
3. Structural model	257.70	86	0.97	0.01	0.04(0.03–0.04)	0.04

**Environment Model**						
1: Configual model	69.76	21	0.96		0.04(0.03–0.05)	0.03
2: Measurement model	74.60	25	0.96	0.00	0.04(0.03–0.05)	0.03
3. Structural model	78.06	28	0.96	0.00	0.04(0.03–0.05)	0.03

Based on the univariate χ^2 ^incremental values (*p *< 0.05)

a: The cut-off points of model fit: CFI greater than .90, Changes in CFI equal or less than 0.01, the point estimate of RMSEA less than 0.05 and the 90%CI of RMSEA less than 0.08, SRMR less than 0.08.

b: Equality constraints related to item Enjoy3 and item Comp3 were removed sequentially

**Table 3:** Tests for Factorial Invariance of the Four Models across Different Age Groups: Goodness-of-Fit Statistics

Model	S-B χ^2^	Df	CFI^a^	Changes in CFI^a^	RMSEA (90%CI)^a^	SRMR^a^
**Enjoyment-Competence Model**						
1: Configual model	138.11	30	0.95		0.05(0.04–0.06)	0.03
2: Measurement model	177.06	37	0.93	0.02	0.05(0.04–0.06)	0.04
Partial measurement model^b^	152.00	34	0.94	0.01	0.05(0.04–0.06)	0.04
3. Structural model	151.80	35	0.94	0.01	0.05(0.04–0.06)	0.04

**Outcome expectation Model**						
1: Configual model	148.87	49	0.95		0.04(0.03–0.05)	0.03
2: Measurement model	206.11	56	0.92	0.03	0.04(0.04–0.05)	0.05
Partial measurement model^c^	172.05	54	0.94	0.01	0.04(0.03–0.05)	0.04
3. Structural model	196.65	55	0.93	0.02	0.04(0.04–0.05)	0.05

**Social Support Model**						
1: Configual model	249.55	76	0.97		0.04(0.04–0.05)	0.03
2: Measurement model	296.44	83	0.96	0.01	0.04(0.04–0.05)	0.04
Partial measurement model^d^	253.31	81	0.97	0.00	0.04(0.03–0.04)	0.03
3. Structural model	281.42	87	0.96	0.01	0.04(0.04–0.05)	0.05

**Environment Model**						
1: Configual model	54.93	20	0.97		0.04(0.02–0.05)	0.03
2: Measurement model	72.25	24	0.96	0.01	0.04(0.03–0.05)	0.03
Partial measurement model^e^	68.13	23	0.96	0.01	0.04(0.03–0.05)	0.03
3. Structural model	104.83	26	0.94	0.03	0.05(0.04–0.06)	0.03

Based on the univariate χ^2 ^incremental values (*p *< 0.05)

a: The cut-off points of model fit: CFI greater than .90, Changes in CFI equal or less than 0.01, the point estimate of RMSEA less than 0.05 and the 90%CI of RMSEA less than 0.08, SRMR less than 0.08.

b: Equality constraints related to item Enjoy3 and item Enjoy5 were removed sequentially

c: Equality constraints related to item Socbel3 and item Socbel4 were removed sequentially

d: Equality constraints related to item Parsupp3 and item Teacher2 were removed sequentially

e: Equality constraints related to item Opp2 were removed

The 'configural invariance' across independent samples of four countries was then examined. As shown in Table 4, the results of the four multi-group analyses for each configural models were satisfactory (CFI > 0.90 and RMSEA < 0.05). The tests of S-B χ^2 ^were all significant (*p *< 0.001), which may be due to sensitivity to large sample size. After that, testing for 'measurement invariance' was conducted. For the perceived enjoyment and perceived competence sub-scales, the pair of error co-variances (Enjoyment "I usually prefer to watch rather than play games" and Competence "I feel really bad when I get out of breath from running around") was also specified for each group. Despite the fact that the four multi-group measurement models revealed satisfactory fit to the data, based on the Byrne's criteria (change in CFI ≦ 0.01), 'partial measurement invariance' was invoked, suggesting that all previously imposed equality constraints remained except those identified to be non-invariant. In the present study, the assumptions of using partial measurement invariance were met. The non-invariant parameters in the four measurement models were identified using LM Test (incremental univariate χ^2 ^< 0.05). Also evident in Table 4, after re-specification of the non-invariant parameters, the three partial measurement models yielded satisfactory results aside from the perceived social-physical environment sub-scales (Change in CFI = 0.03), which could not be allowed to free further parameters for estimation given the violation of the partial measurement assumptions. Finally, the results of the 'structural invariance' for the four models were all satisfactory except for the perceived social-physical environment sub-scales (Change in CFI = 0.04), which revealed that this multi-group invariance model underwent some extent of deterioration. Hence, overall, except the perceived social-physical environment sub-scales, the factorial invariance of all remaining measures across four culturally different countries, gender and two age groups was demonstrated. As revealed in table 5, zero-order correlation analysis of physical activity influences based on computed mean scores of the latent variables generally showed an expected pattern of positive relationships within and between the measures of the perceived personal, social and social-physical environmental correlates.

**Table 4:** Tests for Factorial Invariance of the Four Models across Four European Countries: Goodness-of-Fit Statistics

Model	S-B χ^2^	Df	CFI^a^	Changes in CFI^a^	RMSEA (90%CI)^a^	SRMR^a^
**Enjoyment-Competence Model**						
1: Configual model	160.81	64	0.96		0.04(0.03–0.05)	0.04
2: Measurement model	318.88	86	0.91	0.05	0.05(0.05–0.06)	0.07
Partial measurement model^b^	229.22	83	0.95	0.01	0.04(0.04–0.05)	0.04
3. Structural model	233.66	86	0.95	0.01	0.04(0.04–0.05)	0.05

**Outcome expectation Model**						
1: Configual model	234.80	95	0.93		0.04(0.04–0.05)	0.04
2: Measurement model	315.52	119	0.91	0.02	0.05(0.04–0.05)	0.06
Partial measurement model^c^	280.04	113	0.92	0.01	0.04(0.04–0.05)	0.05
3. Structural model	297.20	116	0.92	0.01	0.04(0.04–0.05)	0.06

**Social Support Model**						
1: Configual model	261.03	152	0.98		0.03(0.02–0.04)	0.03
2: Measurement model	344.88	173	0.96	0.02	0.03(0.03–0.04)	0.04
Partial measurement model^d^	294.62	167	0.98	0.00	0.03(0.03–0.04)	0.04
3. Structural model	374.84	182	0.97	0.01	0.04(0.03–0.04)	0.06

**Environment Model**						
1: Configual model	68.91	44	0.98		0.03(0.01–0.04)	0.03
2: Measurement model	123.33	56	0.95	0.03	0.04(0.03–0.05)	0.04
Partial measurement model^e^	111.70	53	0.95	0.03	0.04(0.03–0.05)	0.04
3. Structural model	135.28	62	0.94	0.04	0.04(0.03–0.05)	0.05

Based on the univariate χ^2 ^incremental values (*p *< 0.05)

a: The cut-off points of model fit: CFI greater than 0.90, Changes in CFI equal or less than 0.01, the point estimate of RMSEA less than 0.05 and the 90%CI of RMSEA less than 0.08, SRMR less than 0.08.

b: Equality constraints related to item Enjoy 4 and item Enjoy3 were removed sequentially

c: Equality constraints related to item Socbel 2 and item Socbel 3 were removed sequentially

d: Equality constraints related to item Parsupp 3 and item Friend 2 were removed sequentially

e: Equality constraints related to item Opp 2 was removed

**Table 5:** Inter-correlations between perceived personal, social and environmental correlates and alpha estimates

*Correlates*	1	2	3	4	5	6	7	8	9	10	11	α
1. Enjoyment	-	0.30**	0.17**	0.21**	0.31**	0.17**	0.28**	0.32**	0.09**	0.01	0.12**	0.57 (0.51–0.62)
2. Perceived competence		-	0.16**	0.09**	0.18**	0.01	0.16**	0.17**	0.16**	-0.08**	-0.05**	0.33 (0.30–0.41)
3. Parental support			-	0.37**	0.37**	0.26**	0.17**	0.12**	0.10**	0.11**	0.15**	0.63 (0.57–0.67)
4. Parental encouragement				-	0.31**	0.27**	0.16**	0.17**	0.08**	0.02	0.12**	0.50 (0.45–0.53)#
5. Social support from friends					-	0.29**	0.24**	0.16**	0.20**	0.06**	0.13**	0.76 (0.73–0.79)
6. Teacher social support						-	0.12**	0.05*	-0.05	0.15**	0.14**	0.68 (0.63–0.67)
7. Social outcome expectations							-	0.48**	0.09**	0.10**	0.10**	0.55 (0.44–0.65)
8. Functional outcome expectations								-	0.12**	-0.01*	0.09**	0.58 (0.52–0.66)
9. Opportunity									-	-0.16**	0.02	0.44 (0.12–0.57)
10. Facility										-	0.22**	0.20 (0.08–0.29)#
11. Licence											-	0.45 (0.25–0.54)#

Note: ** Correlation is significant at the 0.01 level (2-tailed) *Correlation is significant at the 0.05 level (2-tailed) α range between countries in parenthesis # inter-correlations for two-item measures

### Reliability further estimates of validity

Reliability estimates for all sub-scales were performed initially on the four counties independently and then on the pooled data set. Alpha estimates for the sub-scales within the pooled data as well as estimates within each the four countries are shown in Table 5. Alpha estimates were variable, but generally above 0.50 for most sub-scales. Except for the *Facility *sub-scale, two item sub-scales showed evidence of satisfactory inter-correlation.

Results for the MANOVA (see Table 6) show a significant main effect for stage of change for exercise by the perceived personal, social and environmental correlates (Wilk's lambda = 0.79, *F *(44,14076) = 20.40, p < 0.001). Follow-up ANOVAs showed that, except for Licence (*F*(4,3689) = 1.71, p > 0.05), the mean scores on all correlates differed by stage of physical activity behaviour change. These included perceived enjoyment (*F*(4, 3689) = 100.64, p < 0.001), perceived competence (*F*(4, 3689) = 99.62, p < 0.001), perceived social support from parents (*F*(4, 3689) = 41.75, p < 0.001), perceived parental encouragement (*F*(4, 3689) = 12.39, p < 0.001), perceived social support from friends (*F*(4, 3689) = 64.50, p < 0.001), perceived teacher support (*F*(4, 3689) = 4.29, p < 0.01), social outcome expectations (*F*(4, 3689) = 23,19, p < 0.001), functional outcome expectations (*F*(4, 3689) = 44,31, p < 0.001), perceived opportunity (*F*(4, 3689) = 29.43, p < 0.001), and perceived facility access (*F*(4, 3689) = 3.60, p < 0.001). With the exception of perceived teacher support, licence and facility, post hoc Scheffe's analysis generally indicated patterns of significantly increasing mean scores by advancing stage of behaviour change for correlates. With respect to facility however, a significantly lower score was revealed for those in the maintenance stage as compared to those in the pre-contemplation stage.

**Table 6:** Means, standard deviations, *P *values and Sheffe's post-hoc comparison test results on perceived personal, social, and environmental correlates by stage of physical activity behaviour change

	Stage of Physical Activity Behaviour Change					
	Pre-contemplation (n = 104) M (SD)	Contemplation (n = 215) M (SD)	Preparation (n = 1575) M (SD)	Action (n = 273) M (SD)	Maintenance (n = 1527) M (SD)	*P*
***Personal correlates***						
Enjoyment	2.10 (0.52)	2.26 (0.48)	2.38 (0.40)	2.53 (0.37)	2.59 (0.34)	< 0.001^a^
Perceived competence	1.97 (0.48)	2.06 (0.48)	2.11 (0.43)	2.25 (0.42)	2.38 (0.41)	< 0.001^b^
Social outcome expectations	2.30 (0.47)	2.36 (0.47)	2.37 (0.41)	2.45 (0.44)	2.49 (0.39)	< 0.001^c^
Functional outcome expectations	2.57 (0.45)	2.65 (0.36)	2.70 (0.32)	2.75 (0.32)	2.80 (0.28)	< 0.001^d^
***Social correlates***						
Parental support	1.44 (0.60)	1.35 (0.58)	1.32 (0.47)	1.48 (0.61)	1.56 (0.58)	< 0.001^e^
Parental encouragement	1.95 (0.84)	1.91 (0.84)	1.93 (0.77)	2.16 (0.87)	2.11 (0.88)	< 0.001^f^
Social support from friends	1.98 (0.77)	1.86 (0.83)	1.93 (0.70)	2.22 (0.82)	2.32 (0.73)	< 0.001^g^
Teacher social support	1.60 (0.65)	1.63 (0.65)	1.52 (0.55)	1.65 (0.67)	1.58 (0.64)	0.002*
***Environmental correlates***						
Opportunity	2.46 (0.52)	2.50 (0.54)	2.52 (0.49)	2.60 (0.47)	2.69 (0.45)	< 0.001^h^
Facility	1.51 (0.57)	1.46 (0.56)	1.40 (0.51)	1.44 (0.56)	1.37 (0.52)	< 0.01^i^
Licence	2.46 (0.63)	2.30 (0.69)	2.34 (0.65)	2.36 (0.66)	2.37 (0.64)	0.144

**a**) precon/con/prep<act/maint; precon/con<prep; preco<con. **b**) precon/con/prep<act/maint; act<maint. **c**) precon<act/maint. **d**) precon<prep/act/maint; con<maint **e**) con/prep<maint. **f**) con/prep<act. **g**) precon/con/prep<act/maint. **h**) precon/con<act/maint. **i**) precon>maint

*Note: No significant differences revealed through Sheffe's post-hoc test for teacher social support

An independent sample t-test was used to compare those young people categorised as high versus low physical activity based on whether they achieved 60 minutes of moderate activity derived from the actigraph data. Significantly higher mean levels of perceived enjoyment (M = 2.49 versus 2.39, 95%CI -0.14/0.06; *t *= -5.11; p < 0.001), perceived parental support (M = 1.51 versus 1.32. 95%CI -0.25/-0.14; *t *= -2.87 p < 0.001); perceived friend support (M = 2.25 versus 1.84, 95%CI -0.49/-0.35; *t *= -5.69; p < 0.001); perceived teacher support (M = 1.55 versus 1.37, 95%CI -0.24/-0.12; *t *= -3.42; p < 0.001), and perceived access to facilities (M = 1.47 versus 1.30, 95%CI -0.21/-0.11; *t *= -2.33; p < 0.05) were evident for those who achieved versus not achieved at least 60 minutes of accumulated moderate physical activity on at least three days. There were no significant differences between the two physical activity groups for perceived competence (M = 2.24 versus 2.25, 95%CI -0.03/-0.05; *t *= 0.46; p > 0.05), social outcome expectations (M = 2.44 versus 2.41, 95%CI -0.07/0.01; *t *= -1.56; p > 0.05), functional outcome expectations (M = 2.73 versus 2.73, 95%CI -0.03/0.02; *t *= -0.41; p > 0.05), perceived parental encouragement (M = 2.01 versus 1.94, 95%CI -0.15-0.02; *t *= -1.58; p > 0.05) and licence (M = 2.42 versus 2.38, 95%CI -0.10/0.19; *t *= -1.33; p > 0.05). In the case of opportunity, a lower mean score (M = 2.60 versus 2.65, 95%CI -0.00/-0.10; *t *= 2.10; p < 0.05) was found for the high versus low physical activity group.

## Discussion

This study used data from nine and fifteen year old boys and girls from four European countries to examine the factorial validity and invariance of a computerised inventory measuring theoretically derived and previously supported influences of physical activity in young people. We also examined further construct validity of the measures in relation to both a self report measure and an objective measure of physical activity.

### Factorial validity and gender, age-group and cross-cultural invariance

The factorial validity of all the four baseline measurement models of perceived personal, social and social-physical environmental correlates of physical activity within the computerized inventory was established. More specifically, acceptable fit indices were found for the sub-scales comprising a) perceived enjoyment and perceived competence, respectively; b) sub-scales pertaining to social and functional outcome expectations, c) sub-scales of perceived parental support, perceived parental encouragement, peer support and teacher support, respectively and d) the sub-scales regarding perceived social-physical environment (i.e. opportunity, license and facility). Based on invariance estimates with respect to age groups, results further revealed slight differences between the 9 and 15 year olds, particularly in relation to the sub-scales reflecting perceptions of the social-physical environment regarding physical activity. Findings may reflect that understanding of environmental barriers and enabling factors and their resulting perceived influence on physical activity may differ between younger and older adolescents. This may be due to the major shifts that often occur around this age, such as movement to a different school and greater child autonomy or parental license [36].

Only the cross cultural factorial invariance of the sub-scales pertaining to the perceived physical-social environment were not supported (Model 4). These findings may reflect that items pertaining to opportunity, facility access and license may convey different meanings for people in diverse socio-cultural contexts because responses are interpreted according to cultural specific norms leading to differences in meaning and understanding of particular items [35]. Hence, discrepant cross-cultural responses may be lead to construct or item bias.

The sub-scales embedded in Models 1, 2 and 3 showed good evidence of cross-cultural factorial invariance. These results complement previous findings [13,15] and suggest that the measures of perceived enjoyment, perceived competence, social and functional outcome expectations as well as the measures of perceived parental social support and encouragement, perceived peer support and support from teachers generally may be used with confidence in multi-centre studies with children and adolescents even in diverse cultural samples. As we observed in our initial exploratory factor analysis, parental support and encouragement has also been found to represent a one-dimensional construct [e.g. [18]]. In the present case, however, this measure showed evidence of good fit when modified and split into two separate dimensions in the confirmatory factor analysis. Indeed, the semantics of the items representing perceived parental support and encouragement, respectively, reflect two types of social support. First, perceived social support in the form of *social encouragement *seems embedded in the two following items (e.g. "How often does your mum or dad tell you to exercise or play sports?"; "How often does your mum or dad tell you that exercise is good for your health?"). Second, social support in the form of *social participation*, embracing overt social support like playing with the child and "gatekeeper" or instrumental support [37,38] seems embedded in the following three items (e.g. "How often does your mum or dad take you to exercise or play sports?"; "How often does your mum or dad watch you take part in exercise or play sports?"; "How often does your mum or dad exercise or play sports with you?"). These findings are in line with research that has taken a multidimensional approach to the concept of social support, focusing on for example adult modeling, support (e.g. adults are physically active with me) as well as transport and encouragement support [e.g. [14,38]]. Our results extend recent findings [e.g. [37]] by showing evidence of cross-cultural factorial invariance for different types of perceived social support as reflected in our two-dimensional measure.

In line with previous studies [e.g. [6]], *outcome expectations *was also a-priori forwarded as a one-dimensional construct. However, in this sample, outcome expectations also showed evidence of good fit for two separate dimensions in the confirmatory factor analysis. A closer inspection of the items reveals some which are more social in nature pertaining to gaining friendship and having fun, whereas others are functional in nature pertaining to the possibility of obtaining skills, physical fitness and additional health gains by being physically active. Our findings support work which has dichotomized outcome expectations into functional and social ones [39]. Further, the two dimensions of outcome expectations we obtained seem to capture what young people have reported unprompted when asked to complete a free-response format questionnaire to elicit relevant expectations regarding outcomes related to physical activity [18]. Hence, the two dimensions of outcome expectations generated through the confirmatory factorial analysis hold promise for being incorporated into others studies to represent a more differentiated set of outcome expectations as influences on youth physical activity.

Traditionally, the least studied domain of potential determinants of youth physical activity is the perceived social-physical environment for physical activity [18]. The social aspects of such influences go beyond social reinforcement of significant others (e.g. modelling, support and encouragement), and pertain to the physical environment that either facilitates or creates social and/or physical barriers towards young peoples' physical activity. Recent work has increased our understanding of the role of the environment in relation to physical activity [e.g. [40]] and confirmed that factors such as facility access and time outdoors are consistently associated with children's physical activity [28]. However, results related to perceptions of the environment and physical activity, such as safety and access to playmates in the neighbourhood are less consistent [40,41]. In the present study we sought to build on available evidence by examining a wider range of potential influences alongside perceived environmental influences typically examined in previous research. These included perceived social and physical opportunities for being active, including accessibility of playmates, access to facilities and playgrounds, safety in the neighbourhood environment, licence from parents to play out, and parental restrictions when outside of the home. We did not forward any a-priori expectations as to the potential dimensionality of these different facets of the physical-social environment. However, the fit indices for the baseline confirmatory factor analysis models indicated that perceived physical-social environmental influences seem best captured as three distinct aspects of the environment, labelled *opportunity*, *facility *and *licence*, respectively. These findings parallel meta-analytical ones among adults [42] and complement early research whereby measures of the physical- social environment were captured by single items [18]. However, the perceived *opportunity*, *facility *and *licence *measures need further refinement in order to show factorial validity and invariance across age groups and cross-culturally diversified samples. Work such as that by Timperio et al. [43] point to interpreting perceptions of the environment in relation to specific behaviours such as active travel rather than more global physical activity used in this study.

### Reliability and further estimates of validity

Internal reliability estimates and inter-item correlations varied considerably for the sub-scales pertaining to perceived environmental influences and for the perceived competence sub-scale. In some instances, reliability estimates were below the level usually considered acceptable cut-off level of 0.70 [44]. However, several estimates were in line with those found for similar measures in previous studies which also show reliabilities ranging from 0.48 to 0.88 [6,14,45]. Nevertheless, further refinement of these sub-scales seems warranted. In particular, further work on the development of the perceived competence sub-scale and the items making up the measures pertaining to perception of opportunities, facility access and licence, respectively should be given priority in order to raise the alpha values. Indeed, the low inter-correlation between the two items making up the perceived facility sub-scale may reflect that these two items may not be measuring a single construct of "facility", but rather aspects of two different environments (having playgrounds at school versus having these near at home). It is possible that low alpha values obtained for some sub-scales may have resulted from a small number of items per scale [44]. Hence, future work should also aim to generate a larger initial pool of items to reflect differential aspects of the perceived social-physical environment and perceived competence balanced by the need to keep the number of items in an inventory manageable by the target age group.

The rather low alpha value observed for the perceived competence sub-scale is unusual and warrants further investigation. Nevertheless, perceived competence and enjoyment as sub-scales in measurement model 1 was correlated in line with expectations [6,46,47] and was systematically related to stage of change in the expected direction (i.e. greater perceived competence in the latter stages of change). Functional and social outcome expectations as well as perceptions of peer support, parental encouragement and parental support were also greater in the latter stages of change. Further, those in the activity group with levels of daily physical activity in line with current guidelines reported greater levels of enjoyment, peer support, parental support and access to facilities than those not accumulating on average 60 minutes of at least moderate activity on measurement days. Increments in mean values of the perceived personal, social and social-physical environmental influences between stages of change should be considered modest in size. Nevertheless, the total pattern of results for the correlates tested against self-reported and objective indices of physical activity add further support to their construct validity by indicating they can differentiate between young people with different levels of physical activity.

To some extent analysis of construct validity of the measures of teacher support and the environmental influences are not in line with expectations and are potentially conflicting. No difference for perceived teacher support across stages of exercise behaviour change may reflect that teachers are generally supportive of all students, irrespective of their level of exercise and physical activity. Further, with respect to perception of environmental influences, enhanced opportunities for play related in the expected direction to stages of change for exercise, but this was not the case for facility and license. In terms of facilities, one could speculate whether those most physically active participate in organized sports or exercise contexts [48], where facilities are not necessarily near their home environment. However, in contrast, those in the high physical activity group based on the actigraph data reported higher perceived teacher support and better access to facilities than those in the low activity groups.

### Conclusions and future research

In general, the confirmatory factor analyses provided evidence for the factorial validity of the four measurement models. Additional analyses revealed that, except for the perceived physical-social environment measure, invariance of all measures were obtained across four culturally different countries, genders and two age groups. For most measurements, additional evidence of construct validity was also obtained. Possibilities for future research include further development of the perceived competence sub-scale to enhance reliability and further development of the sub-scales pertaining to opportunities, facilities and licence to obtain age-specific and cross-cultural invariance [40]. If successful, future research could make use of the current measures to examine their influence on physical activity behaviour in other samples using predictive designs. Longitudinal data is important to test whether changes in physical activity over time are related to changes in the measurements described. The measures also hold promise for inclusion in cross-cultural multi-centre intervention studies to examine mediated intervention effects of the current correlates on physical activity change. Despite these limitations, to our knowledge this is the first study to demonstrate simultaneous country, age and gender invariance in a wide range of sub-scales in a large culturally diverse sample.

## Competing interests

The authors declare that they have no competing interests.

## Authors' contributions

AP and ARC developed and pilot tested the initial computerised questionnaire on British school children and developed the protocol for its use in the European Youth Heart Study. P-WK contributed to the analyses. YO drafted the initial manuscript and contributed to the analyses. All authors contributed to the writing of the manuscript and read and approved the final submitted version.
